# Marchiafava-Bignami Disease: A Rare Association With Dysdiadochokinesia and Ataxic Gait

**DOI:** 10.7759/cureus.41492

**Published:** 2023-07-07

**Authors:** Mahmoud Abouibrahim, Ansh Agarwal, Ugochinyere Ottih, Kapilraj Ravendran, Galaxy Bista, Mansoor Zafar, Garabedyan Hovagim, Kadir Hacikurt

**Affiliations:** 1 Internal Medicine, Conquest Hospital, East Sussex Healthcare NHS Trust, St. Leonards-on-Sea, GBR; 2 General Surgery, East Sussex Healthcare NHS Trust, Brighton and Hove, GBR; 3 Medicine, Gradscape, London, GBR; 4 Medical School, Medical University of Sofia, Sofia, BGR; 5 Gastroenterology, General Internal Medicine, Conquest Hospital, East Sussex Healthcare NHS Trust, St. Leonards-on-Sea, GBR; 6 Neurology, Conquest Hospital, East Sussex Healthcare NHS Trust, St. Leonards-on-Sea, GBR; 7 Radiology, Conquest Hospital, East Sussex Healthcare NHS Trust, St. Leonards-on-Sea, GBR

**Keywords:** magnetic resonance imaging, corpus callosum, ataxic gait, dysdiadochokinesia, marchiafava-bignami disease

## Abstract

Marchiafava-Bignami disease (MBD) is a rare neurological disorder characterized by demyelination and necrosis of the corpus callosum. The non-specific signs and symptoms associated with MBD including dysarthria, impaired walking, pyramidal signs, primitive reflexes, seizures, incontinence, sensory symptoms, gaze palsies, and altered mental state result in a challenging diagnosis. Here, we report the case of a 64-year-old female presenting with dizziness, gait ataxia, and a history of recurrent falls for several months. Initial blood tests indicated anaemia, hypokalemia, hypomagnesemia, and mildly elevated inflammatory markers. Her presentation was initially attributed to a multifactorial aetiology, including a urinary tract infection, orthostatic hypotension, and electrolyte imbalances; however, on correction of reversible causes, her symptoms persisted. Moreover, further examination revealed right-hand dysdiadochokinesia. Subsequent brain MRI revealed fluid-attenuated inversion recovery hyperintensity within the corpus callosum and a right-sided pericallosal white matter hyperintensity. Neuro-radiology multidisciplinary team reported these findings consistent with MBD. Management with vitamin B supplementation was promptly initiated alongside alcohol cessation advice. She was also reviewed by physiotherapy teams. This case adds to the paucity of literature on MBD.

## Introduction

Marchiafava-Bignami disease (MBD), first described by pathologists Ettore Marchiafava and Amico Bignami in 1903, is a rare neurological disorder characterized by demyelination and necrosis of the corpus callosum [1,2].

Although known to be common in chronic alcoholics and those with malnutrition, its diagnosis can be challenging due to vague and non-specific clinical presentation. MBD patients have reportedly presented with dysarthria, impaired walking, pyramidal signs, primitive reflexes, seizures, incontinence, sensory symptoms, gaze palsies, and altered mental state including confusion, delirium, unconsciousness, impaired memory, and disorientation [3]. The gold standard for diagnosis involves visualisation of the lesions in the corpus callosum via magnetic resonance imaging (MRI) [4].

This case report presents the clinical details and management of a patient presenting with ataxia and diagnosed with MBD, with the goal of contributing to the growing body of literature surrounding this rare disease.

## Case presentation

A 64-year-old female patient was admitted to the emergency department (ED) due to symptoms of dizziness, an unsteady gait, and a history of recurrent falls. Upon reviewing her medical records, it was observed that these symptoms were persistent over an extended period resulting in two prior hospital admissions within the past six months.

The patient’s medical history revealed several comorbidities, including atrial fibrillation, hypertension, type 2 diabetes mellitus, iron deficiency, osteoporosis, depression, and a history of excessive alcohol consumption. An ataxic gait was observed during the examination, with no signs of weakness. Blood tests indicated the presence of anaemia, electrolyte imbalances characterized by hypokalaemia and hypomagnesemia, and slightly elevated inflammatory markers (Table 1).

**Table 1:** Blood test results.

Investigation	Day one	Day six	Unit of measurement	Reference range
Haemoglobin	105	76	g/L	125–165
White cell count	7.9	6.94	10^9^/L	4.0–11.0
Platelet count	359	337	10^9^/L	150–400
Haematocrit	0.287	0.226	-	0.37–0.47
Mean cell volume	83.9	89.7	fl	80–100
Red cell count	3.42	2.52	10^12^/L	3.8–5.8
Mean cell haemoglobin	30.7	30.2	pg	27.0–32.0
Red cell distribution width	15.8	15.2	%	11.8–14.8
Nucleated red blood cell	0	0	10^9^/L	0–0.01
Neutrophils	6.19	4.89	10^9^/L	2–7.5
Lymphocytes	0.99	1.58	10^9^/L	1.50–4.00
Monocytes	0.67	0.37	10^9^/L	0.2–0.8
Eosinophils	0.01	0.08	10^9^/L	0–0.4
Basophils	0.04	0.02	10^9^/L	0–0.1
Serum sodium	134	136	mmol/L	133–146
Serum potassium	2.1	4.4	mmol/L	3.5–5.3
Serum TCO_2_	46	-	mmol/L	22–29
Serum urea	5.9	3.8	mmol/L	2.5–7.8
Serum creatinine	117	74	µmol/L	45–84
Serum bilirubin	20	4	µmol/L	0–21
Serum alkaline phosphatase	101	72	U/L	30–130
Serum alanine transaminase	18	10	U/L	10–35
Serum albumin	40	29	g/L	35–50
Serum calcium	2.28	-	mmol/L	-
Serum magnesium	0.53	-	mmol/L	0.7–1.0
Serum C-reactive protein	52	-	mg/L	0–5
Estimated glomerular filtration rate (CKD-EPI)	42	74	mL/minute/1.73m^2^	-
Erythrocyte sedimentation rate	-	42	mm/h	3–15
Reticulocyte count	-	72.3	10^9^/L	10–100
Serum total protein	-	49	g/L	60–80
Serum immunoglobulin G	-	5.6	g/L	7.00–16.00
Serum immunoglobulin A	-	2.23	g/L	0.70–4.00
Serum immunoglobulin M	-	0.4	g/L	0.40–2.30
Serum C3	-	1.17	g/l	0.90–1.80
Serum C4	-	0.3	g/L	0.10–0.40
Anti-nuclear antibody	-	Negative		
Anti-myeloperoxidase antibody	-	<0.2	IU/mL	0–3.4
Anti-proteinase 3	-	<0.2	IU/mL	0–1.9
Serum vitamin B12	-	515	ng/L	197–771
Serum folate	-	4.7	µg/L	2.4–17.5
Faecal test for haemoglobin	-	12	µg/g	0–9

**Figure 1:**
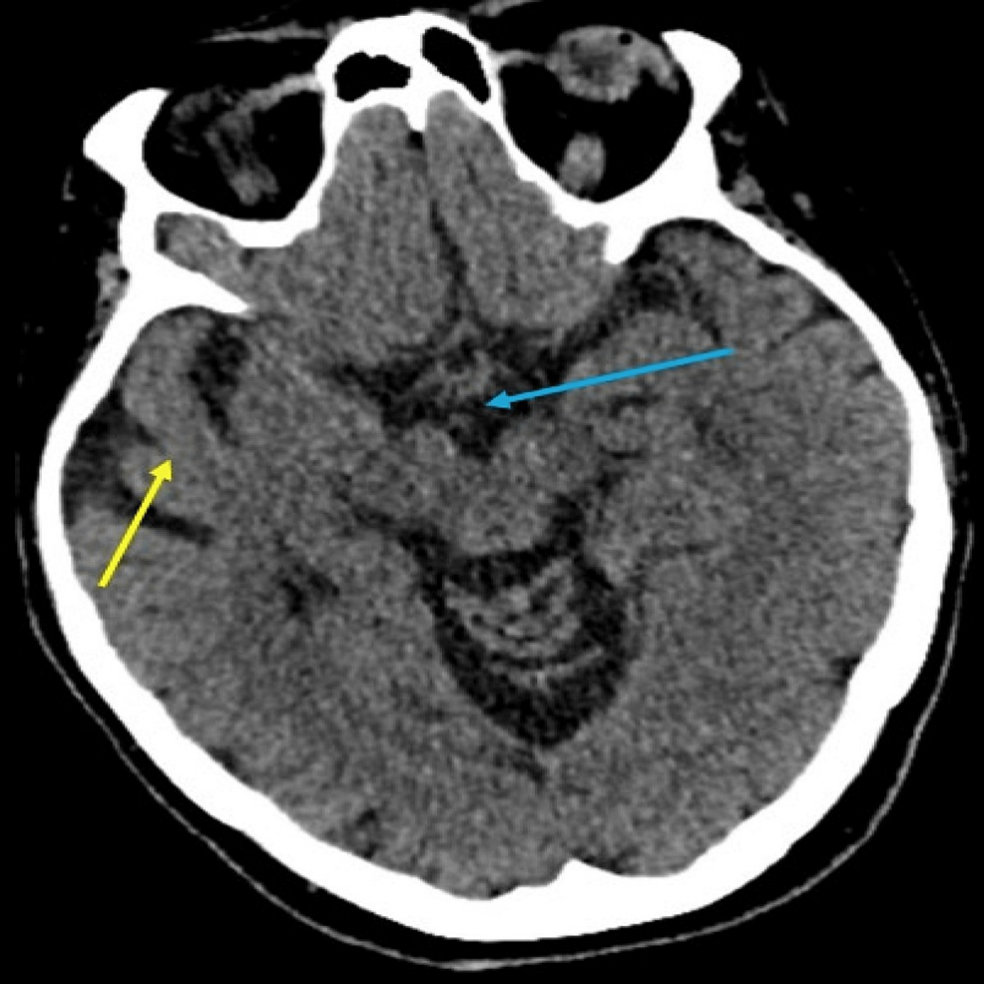
Chronic small vessel disease, mild cerebral atrophy (yellow arrow), and enlargement of cerebrospinal fluid spaces (blue arrow). No acute intracranial pathology can be seen.

Initially, the symptoms were attributed to multifactorial aetiology, including a urinary tract infection, orthostatic hypotension, and electrolyte imbalances. Following treatment with antimicrobials and electrolyte replacement, it was observed that the ataxia did not show any improvement. Through a more detailed patient history, it became apparent that she consumed one bottle of vodka per day. Additionally, upon conducting further examinations, it was noted that she exhibited dysdiadochokinesis in her right hand. These findings prompted the medical team to arrange a computed tomography (CT) scan of the head that showed chronic small vessel disease, mild cerebral atrophy, and enlargement of cerebrospinal fluid spaces with no acute intracranial pathology (Figure 1).

**Figure 2:**
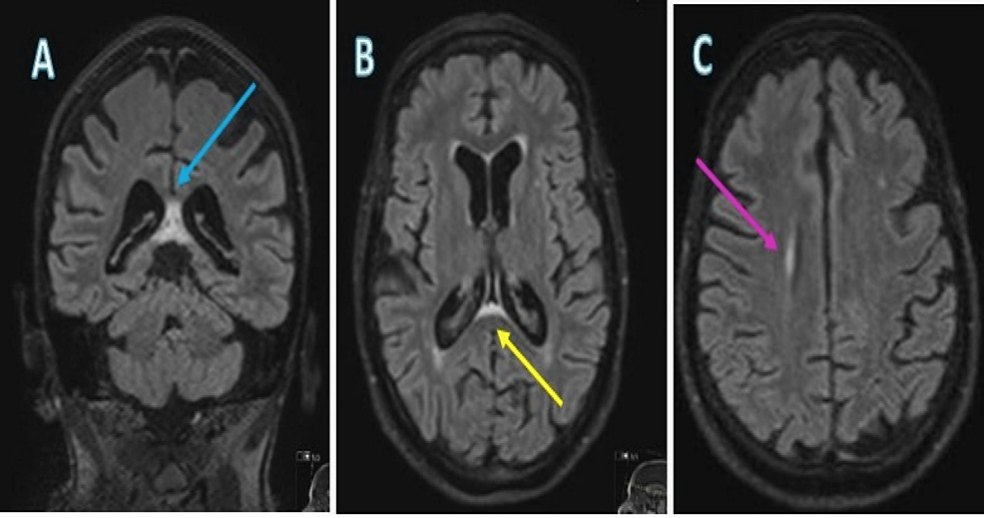
(A) Magnetic resonance imaging of the head coronal and axial fluid-attenuated inversion recovery images revealing hyperintensities within the corpus callosum (blue arrows). (B) Hyperintensities affecting the splenium and extending into the fornix (yellow arrows). (C) Non-specific white matter hyperintensities within the corona radiata (pink arrows).

Subsequently, an MRI of the brain was requested, revealing the presence of fluid-attenuated inversion recovery hyperintensity within the corpus callosum, specifically affecting the splenium and extending to the fornix. Furthermore, a pericallosal white matter hyperintensity was identified on the right side (Figure 2).

These results were discussed in a neuro-radiology meeting, and it was concluded that there is evidence of demyelination involving the splenium of the corpus callosum with marked cerebellar atrophy, consistent with MBD. Consequently, the recommendation was made to initiate vitamin B supplementation, advise cessation of alcohol intake, and refer the patient to rehabilitation for physiotherapy with graded exercises aimed at improving mobility.

## Discussion

MBD is a neurological disorder characterized by demyelination primarily affecting the corpus callosum. The condition was initially described by two Italian pathologists who identified its pathological features during autopsies of three patients. These individuals had a history of chronic alcoholism and were notable for their consumption of substantial quantities of red wine. Initially, the patients experienced status epilepticus, which was followed by the onset of coma [5].

Heinrich et al. found that individuals hospitalized with MBD had a mean age of 46 years and were mostly male (75%). They commonly experienced cognitive impairment (100%), limb hypertonia (54%), dysarthria (50%), and disconnection syndromes (56%). The incidence of seizures and symptoms of the pyramidal tract was less common. The diagnosis of MBD poses a challenge due to the absence of a distinctive clinical profile, making early diagnosis difficult. MBD presents with a range of subtle initial manifestations. These include impaired consciousness, emotional and psychotic symptoms, depressive features, apathy, aggressive behaviour, seizures, hemiparesis, ataxia, and apraxia. It is essential to appreciate the heterogeneous nature of these clinical signs to achieve accurate recognition and timely management of MBD [6].

MRI is an indispensable modality for the diagnosis of MBD, providing specific imaging findings. Lesions exhibiting hyperintensity on different MRI sequences, primarily within the corpus callosum, are indicative of inflammatory edematous tissue. Additionally, lesions can manifest in other cerebral regions, including the hemispheric white matter, and basal ganglia. Notably, the presence of extra callosal lesions is predominantly observed in patients with poor prognoses [7]. The utilization of MRI plays a pivotal role in early MBD diagnosis, enabling timely intervention during the acute phase, and contributing to prognostic assessment.

Currently, no standardized treatment protocol exists for MBD due to the scarcity of trials and reported cases. Nevertheless, early diagnosis and intervention involving thiamine, folic acid, and vitamin B complex have shown the potential in expediting and improving the recovery process. Corticosteroids may be employed to reduce inflammation and stabilize the integrity of the blood-brain barrier. It is important to note that MBD carries a high mortality rate, with the acute form often leading to rapid deterioration and death. Surviving patients are often left with significant neurological impairments. However, in some cases where manifestations are less severe, partial or complete recovery may occur intermittently [2].

Despite the fact that MBD was initially associated only with persistent drinking and/or malnutrition, more recent papers have discussed MBD as a paraneoplastic illness or being connected to procedures [3,7,8]. Even in the absence of typical clinical symptoms, early diagnosis of lesions suggesting MBD is now possible due to advancements in contemporary brain imaging methods [9]. As a result, medical professionals can now suspect MBD even in the emergency room and can start considering the best course of action immediately [3].

Alcoholism remains the most common and significant risk factor for MBD, despite no specific nutritional cause having been identified yet. The precise pathologic mechanism of MBD remains unclear [10]. According to certain theories, MBD may also be a symptom of osmotic myelinolysis syndromes [11,12].

In a literature review, Kohler et al. suggested a typical pattern of severe global dementia. This is demonstrated on MRI showing moderate and severe cortical and subcortical atrophy of anterior and posterior callosal regions, respectively. This has been found to be associated with reduced glucose metabolism in the subcortical and mesial frontal regions via positron emission tomography scan [13].

Cytotoxic oedema has occasionally been observed concurrently in other areas of the brain, and it may occur before the onset of callosal necrosis, indicating a poor prognosis [14-18].

Low apparent diffusion coefficients have been observed in studies utilizing MRI diffusion-weighted imaging (DWI), which has been interpreted as supporting the existence of cytotoxic oedema in the corpus callosum. When compared to fluid-attenuated inversion recovery, DWI can detect more extensive callosal lesions in MBD. However, full recovery following thiamine treatment has also been noted, indicating that this is not always the case. For researching the clinical correlates of MBD and the healing process, diffusion tensor imaging may be used [3,14-18].

## Conclusions

MBD is a very rare neurological disorder that can present with a variety of non-specific symptoms. Chronic alcohol abuse, particularly red wine consumption, is linked to the formation of lesions in the corpus callosum. MRI is the preferred imaging choice. However, due to the rarity of the condition, no standardized protocol has been developed yet. Patients can show improvement with thiamine/folate therapy, as observed in this case. Serial MRIs have demonstrated the reversibility of abnormalities, including a reduction in lesion size.
